# It’s Not Lupus This Time! A Case of Worsening Hypothyroidism in a Patient With Nephrotic Syndrome

**DOI:** 10.7759/cureus.25355

**Published:** 2022-05-26

**Authors:** Sonia Iqbal, Wing Y Wan, Natalie E Mitchell

**Affiliations:** 1 Department of Internal Medicine, Brooke Army Medical Center, Fort Sam Houston, USA; 2 Department of Endocrinology, Mike O'Callaghan Military Medical Center, Nellis Air Force Base, USA

**Keywords:** gel levothyroxine, overt hypothyroidism, nephrotic syndrome, liquid levothyroxine, immune complex mediated glomerulopathy, autoimmune thyroiditis

## Abstract

Nephrotic syndrome can result in worsening of existing hypothyroidism in patients requiring thyroid hormone supplementation. The urinary loss of thyroxine-binding globulin, as well as increased gut edema, likely lead to reduced absorption and retention of exogenous thyroid hormone. Here, we present a case of a patient with Hashimoto’s thyroiditis, previously well-controlled on levothyroxine, who developed symptomatic hypothyroidism as a result of newly diagnosed nephrotic syndrome, whose symptoms improved with transition to an alternative formulation of levothyroxine and treatment of her underlying nephrotic syndrome. It is important to consider nephrotic syndrome as a cause of worsening hypothyroidism in a patient on a fixed dose of levothyroxine given the potential morbidity associated with a missed diagnosis and often need for escalation of dosage. There is no standardized therapy for hypothyroidism exacerbated by nephrotic syndrome, but liquid or gel formulations of levothyroxine may be more effective in patients with absorption problems.

## Introduction

There is a well-defined association between nephrotic syndrome and hypothyroidism as cited in the literature. One study showed that in patients with hypothyroidism, minimal change disease was the most common nephrotic syndrome subtype, whereas membranous nephropathy was the common subtype in patients with normal thyroid function and euthyroid sick syndrome [1]. Our particular case is unique as it features the development of autoimmune thyroiditis in the setting of immune complex medicated glomerulopathy in a patient with clinically quiescent systemic lupus erythematosus (SLE). Nephrotic syndrome causes urinary loss of thyroid-binding globulin, albumin, transthyretin, triiodothyronine (T3), and thyroxine (T4) [2]. Patients with functional thyroids are typically able to compensate for the urinary loss of thyroid hormones; however, in patients dependent on exogenous thyroid hormone, overt hypothyroidism may develop [3]. Here, we present a case of a patient with clinically significant and symptomatic hypothyroidism secondary to newly diagnosed nephrotic syndrome requiring adjustment of exogenous thyroid hormone therapy. Early identification of new or worsening of existing hypothyroidism in patients with recently diagnosed nephrotic syndrome is of paramount importance given the potential morbidity associated with a missed diagnosis and potential need for thyroid hormone repletion therapy.

## Case presentation

**Figure 1:**
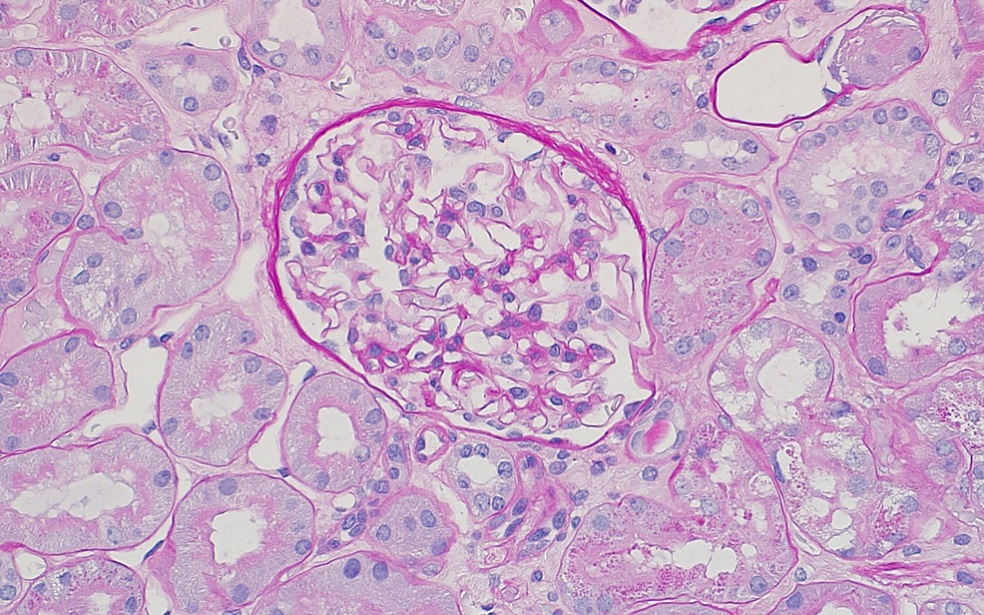
Light microscopy image of the glomerulus showing normal size, architecture, and cellularity of glomeruli with no basement membrane abnormalities.

**Figure 2:**
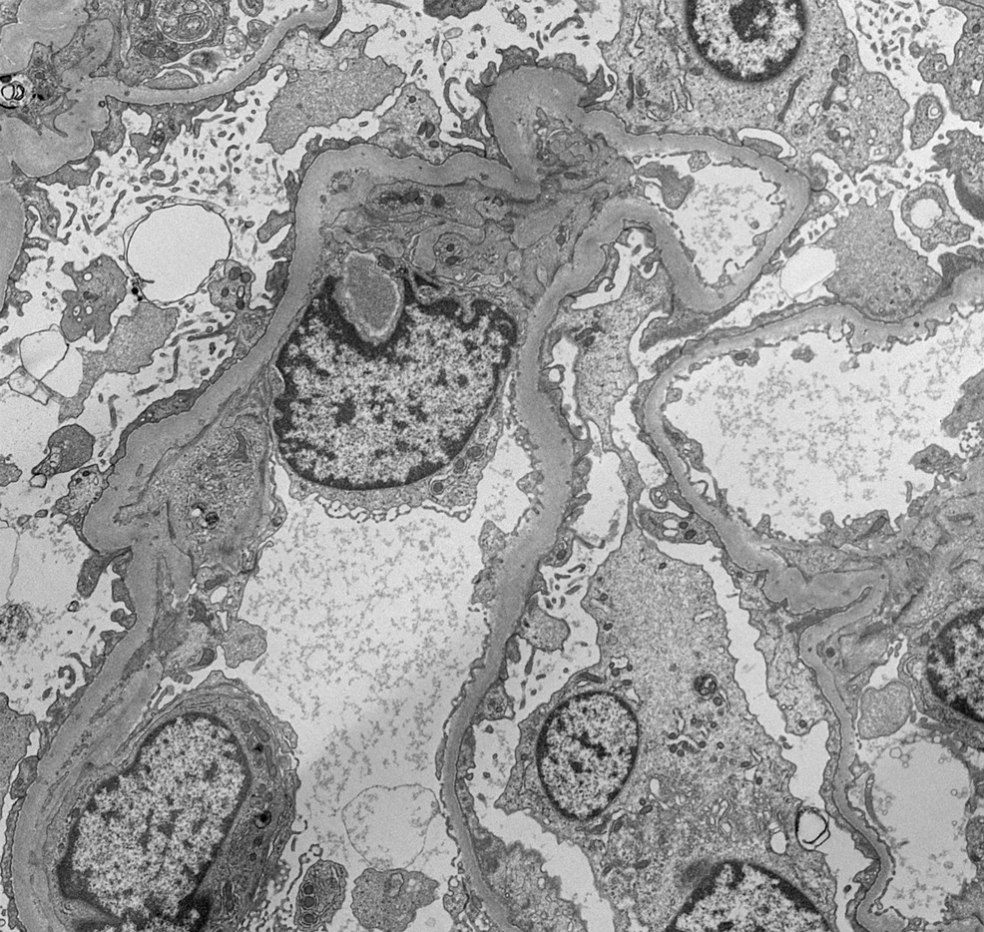
Electron microscopy of the glomerulus showing diffuse podocytopathy with extensive effacement of visceral epithelial cell foot processes and scattered mesangial electron-dense deposits.

A 63-year-old female with a past medical history of hypothyroidism and clinically quiescent SLE presented to the internal medicine clinic with fatigue, weight gain, and new onset bilateral lower extremity pedal edema for three weeks. Prior to this presentation, she had been compliant and well controlled on levothyroxine 100 mcg for approximately two years with thyroid-stimulating hormone (TSH) of 0.570 mcU/mL and T4 of 1.3 ng/dL. Initial laboratory work was notable for new onset nephrotic range proteinuria (protein/creatinine ratio of 5.81 and random total protein of 410 mg/dL), hypoalbuminemia (2.7 g/dL), and hyperlipidemia (total cholesterol of 395 mg/dL and low-density lipoprotein [LDL] of 252 mg/dL) with intact renal function concerning for nephrotic syndrome and severe clinical hypothyroidism (TSH of 121 mcU/mL and T4 of 0.90 ng/dL). Anti-thyroid peroxidase and anti-thyroglobulin antibodies were positive (284 IU/mL and 104 IU/mL, respectively). Antinuclear antibodies, double-stranded DNA antibodies, complement levels, rapid plasma regain screen, hepatitis B, hepatitis C, HIV, urine protein electrophoresis, serum protein electrophoresis, and free light chains were negative. Interventional radiology guided renal biopsy was performed, and pathology was sent out for analysis. Light microscopy, as seen in Figure 1, showed normal size, architecture, and cellularity of glomeruli with no basement membrane abnormalities. Electron microscopy, as shown in Figure 2, showed diffuse podocytopathy with extensive effacement of visceral epithelial cell foot processes consistent with minimal change disease and scattered mesangial electron-dense deposits suggestive of mild immune complex mediated glomerulopathy. Overall, she was given a diagnosis of lupus podocytopathy.

She was referred to nephrology and initiated on high-dose prednisone 60 mg daily for four weeks followed by taper of 50 mg for one week, 40 mg for one week, 30 mg for one week, 20 mg for two weeks, 10 mg for two weeks, 5 mg for two weeks, 2.5 mg for two weeks, and then discontinued. The regimen was selected based on evidence from a case series with patients with lupus podocytopathy that had a rapid response to either glucocorticoid therapy alone or in addition to immunosuppressive agents such as tacrolimus [4]. The patient was referred to endocrinology given significant symptomatic hypothyroidism and was initially bridged with oral liothyronine sodium 5 mcg along with her existing dose of oral levothyroxine 100 mcg while submitting a nonformulary request for gel capsule formation of levothyroxine. After five weeks, liothyronine was discontinued, and levothyroxine sodium 100 mcg (gel capsule) daily was initiated. Repeat TSH was 1.07 mcU/mL, indicating good response to therapy (Table 1). She was ultimately transitioned back to her home levothyroxine dosing following resolution of her nephrotic syndrome with prolonged steroid course.

**Table 1:** Comparison of labs before and after treatment with gel formulation of levothyroxine

	Labs Prior to Treatment	Labs After Treatment	Reference Range
Thyroid-stimulating hormone	121 mcU/ml	1.07 mcU/ml	0.30-5.00
Thyroxine	0.90 ng/dL	-	0.6-1.8
Thyroglobulin antibody	104 IU/mL	-	0-4.1
Thyroperoxidase antibody	284 IU/mL	-	0-5.6
Urine protein/creatinine ratio	5.81	0.11	0-0.14
Albumin	2.7 g/dL	3.9 g/dL	3.5-5.2
Total cholesterol	395 mg/dL	253 mg/dL	<200
Low-density lipoprotein	252 mg/dL	159 mg/dL	60-129

## Discussion

In summary, this patient with worsening hypothyroidism showed improvement after being bridged with liothyronine, likely due to the fast-acting effect of T3, along with tablet levothyroxine and subsequently the gel formulation while the patient was undergoing treatment for nephrotic syndrome. Presently, there is no standardized therapy for hypothyroidism in nephrotic syndrome. It is important to treat given that thyroid hormone replacement therapy has been shown to help preserve or slow the decline of renal function in patients with chronic kidney disease and subclinical hypothyroidism [5]. Gel formulation of levothyroxine was selected because current data suggest that tablet formulation may not be as well absorbed in patients with nephrotic syndrome and treatment with additional hormone is needed for increased efficacy [6]. One case study featured nine patients with hypothyroidism and newly diagnosed nephrotic syndrome, requiring a 17.6% increase in levothyroxine dose [7]. Some experts have suggested adding liothyronine in patients who have an insufficient clinical response to levothyroxine alone; however, this combination has not been proven in efficacy in large clinical trials as of yet [8].

Levothyroxine is available in tablet, gelatin capsule, and liquid formulations, and there is evidence suggestive of better absorption with the latter two formulations [9]. Gelatin capsules are better absorbed during consumption with coffee or proton pump inhibitors (PPIs) and may have been useful in this case where the patient was taking a PPI while on high-dose steroids. Gastrointestinal pathology, including gastroenteritis, celiac disease, bypass surgery, and gut edema, may impair absorption of levothyroxine due to compromised mucosal barrier and raises the question of whether tablet or liquid formulations are more efficacious in patients in certain clinical contexts. One case series featured four patients with hypothyroidism who underwent a Roux-en-Y gastric bypass and achieved normalization of previously elevated TSH levels after switching from oral to liquid levothyroxine [10]. Another case study showed that a patient with autoimmune thyroiditis and gastroenteritis caused by Giardia lamblia was able to achieve a euthyroid state after a switch from oral to liquid levothyroxine [11]. Ultimately, the most important factor in determining efficacy is patient compliance with medication, which can prove challenging with the tablet formulation of levothyroxine if patients do not take them at the appropriate time or take additional medications simultaneously that may affect absorption. One double-blind crossover trial in Italy with 77 patients showed no significant difference between serum TSH, free T4, or free T3 levels regardless of whether liquid formulation of levothyroxine was taken at breakfast or 30 minutes before on empty stomach [12].

While total urinary protein loss was observed in this patient, specific types of proteinuria (i.e., transthyretin, albumin) were not measured. In future studies, this may help elucidate potential associations between hypothyroidism and subcategories of nephrotic syndrome if renal biopsies are inconclusive. One cohort study found an increased risk of hypothyroidism (TSH elevation above 10 mIU/L) in patients with high nonselective urinary protein excretion (>1.75 g/day), and this risk was doubled in those with nephrotic range-proteinuria (>3.5 g/day) [13]. Another study looked at albuminuria in patients with chronic kidney disease without prior thyroid dysfunction and found a negative correlation between the severity of albuminuria and reverse T3 levels but no significant association with other thyroid hormones to include thyroid-binding globulin, TSH, total T3, free T3, and free T4 [14]. Presently, there are not enough data to suggest that this would affect treatment regimens or outcomes.

## Conclusions

The effectiveness and patient agreeability regarding different preparations of levothyroxine is a potential area of further research especially in patients with nephrotic syndrome who subsequently develop or have worsening of hypothyroidism. Additionally, providers should take into account comorbidities that may affect the intake or absorption of tablet levothyroxine and whether liquid levothyroxine would be a better approach for treating the patient. In conclusion, it is important to screen patients with new onset nephrotic syndrome for hypothyroidism so that it may be detected and to recognize that patients with existing hypothyroidism may need to have their therapy adjusted to maintain a euthyroid state.
